# YOLO11-ECA: A Lightweight Tea Bud Detection Method for Complex Tea Plantation Environments

**DOI:** 10.3390/s26175398

**Published:** 2026-08-26

**Authors:** Zhaodong Wang, Xinqiang Liao, Yi Jiang, Hao You, Wenzhi Liao, Yang Li

**Affiliations:** 1 School of Mechanical and Electronic Engineering, Jingdezhen Ceramic University, Jingdezhen 333403, China; xiaopang4129@163.com (Z.W.); l13272868765@163.com (X.L.); 13517984064@163.com (Y.J.); youhao3030@163.com (H.Y.); 2 Research and Innovation Group of Detection Technology and Intelligent Control, School of Mechanical and Electronic Engineering, Jingdezhen University, Jingdezhen 333400, China; 3 Department of Telecommunications and Information Processing, Ghent University, B-9000 Ghent, Belgium; 4 Tea Research Institute, Chinese Academy of Agricultural Sciences, Hangzhou 310008, China

**Keywords:** tea bud detection, YOLO11, efficient channel attention, lightweight object detection, machine vision

## Abstract

Accurate tea bud detection in natural plantation environments is challenged by variable illumination, foliage occlusion, target–background similarity, and dense target distribution. This study proposes YOLO11-ECA, a lightweight detector derived from YOLO11n, to improve the detection performance while reducing the computational demands. The P3/8 prediction branch and its associated high-resolution feature fusion path are removed, while the P4/16 and P5/32 detection scales are retained. Standard adaptive efficient channel attention (ECA) modules are then integrated into the retained feature paths for lightweight channel recalibration. On the validation set, YOLO11-ECA achieved 79.8% precision, 85.1% recall, an 89.2% mAP@0.5, a 52.8% mAP@0.5:0.95, and an F1 score of 82.0%. Relative to YOLO11n, these metrics increased by 1.9, 2.6, 3.1, 0.4, and 2.0 percentage points, respectively, while the parameters and GFLOPs decreased by 7.4% and 28.8%. Scale-specific evaluation showed that P3 removal reduced the AP_*S*_ from 36.0% to 32.4%, whereas adding ECA to the dual-scale architecture increased the AP_*S*_ to 37.0%. On an independent test set, YOLO11-ECA achieved 82.2% precision, an 84.8% mAP@0.5, and an F1 score of 81.0%. The model also reached 86.28 FPS with end-to-end latency of 11.59±0.75 ms under a unified GPU evaluation protocol. These results indicate a favorable balance between tea bud detection performance and computational efficiency.

## 1. Introduction

Tea bud detection provides essential visual information for tea plantation monitoring, yield assessment, harvest planning, and selective mechanical picking. In current tea production, the assessment, counting, and selective harvesting of tender shoots still depend substantially on manual observation, which is labor-intensive and influenced by operator experience. Accurate automated detection can reduce the dependence on manual inspection and provide target information for subsequent counting, localization, and harvesting operations. However, tea buds in natural plantations are commonly distributed among dense foliage and frequently overlap with leaves or neighboring buds. Variations in illumination, viewing angle, canopy structure, and shooting distance further alter their appearance, while the similarity in color and texture between tender shoots and surrounding leaves makes reliable field detection difficult.

Early tea bud recognition methods mainly relied on manually designed color, texture, shape, and threshold-based features. Wei et al. constructed color indices in multiple color spaces to distinguish tender shoots from mature leaves under natural conditions [1]. Wu et al. extracted green characteristics using a color difference operator and applied threshold segmentation to identify tender leaves [2]. These approaches have relatively low computational requirements and can achieve acceptable results under controlled illumination and simple backgrounds. Nevertheless, their performance depends strongly on predefined feature distributions and segmentation thresholds. Changes in natural illumination, weather, viewpoint, and plantation background can alter the color and shape characteristics of tea buds, resulting in incomplete segmentation, background interference, and inaccurate localization. Consequently, handcrafted image processing methods have limited adaptability to complex tea plantation environments.

Convolutional neural networks learn hierarchical visual representations through local connectivity, shared weights, and gradient-based optimization, providing the foundation for modern object detectors [3]. Building on this foundation, semantic segmentation and object detection have gradually become the main approaches to tea bud recognition. Qian et al. developed an encoder–decoder network for pixel-level tea sprout segmentation [4]. Although segmentation can provide detailed target contours, pixel-level annotation requires considerable time and labor. Object detectors directly predict target categories and locations and are therefore more suitable for efficient tea bud localization. Yang et al. improved YOLOv3 for tender shoot recognition and positioning in tea picking applications [5]. Chen et al. combined YOLOv3-based detection with picking point extraction [6]. Xu et al. combined rapid object localization with a classification network to detect and classify tea buds [7]. These studies establish the feasibility of applying deep object detectors to tea bud recognition and localization.

Subsequent research has increasingly focused on detection performance and model efficiency in complex field scenes. Gui et al. introduced lightweight convolution, attention, weighted feature fusion, and an improved bounding box loss into YOLOv5 to reduce the model size and computational cost [8]. Li et al. incorporated Squeeze-and-Excitation attention into YOLOv5m and combined the detector with Kalman filter-based tracking to support tea bud counting in natural image sequences [9]. Xie and Sun integrated deformable convolution, attention, and improved spatial pyramid pooling into YOLOv8s to strengthen tea bud feature extraction under complex backgrounds [10]. Beyond task-specific detector redesign, architecture-independent methods such as frequency regularization have also been investigated to reduce information redundancy in convolutional neural networks [11]. These studies demonstrate the value of lightweight convolution, attention, feature fusion, and redundancy control. However, their detection-scale configurations are generally inherited from general-purpose architectures, and the necessity of each prediction branch for a specific tea bud target distribution has received limited analysis.

Different prediction branches operate on feature maps of different resolutions and therefore impose different computational costs. A high-resolution branch preserves detailed spatial information, but its associated fusion and prediction operations also increase the inference demand. Whether all inherited detection scales are necessary for a particular tea bud dataset should therefore be determined experimentally rather than assumed from a general-purpose architecture. Moreover, representative tea bud studies have mainly reported the AP or mAP at an IoU threshold of 0.5 together with the parameter count, FLOPs, or detection speed. These indicators alone do not fully characterize localization performance under stricter IoU thresholds, scale-dependent detection behavior, performance on images excluded from model development, or end-to-end single-image inference efficiency.

To address these issues, a lightweight tea bud detector named YOLO11-ECA is developed from YOLO11n. Based on the target-scale characteristics of the dataset, the P3/8 prediction branch and its associated high-resolution feature fusion path are removed, while the P4/16 and P5/32 detection scales are retained. Standard adaptive efficient channel attention (ECA) modules are incorporated into the retained P4 and P5 feature paths to recalibrate channel responses with minimal additional parameters and computation. The effects of detection scale adjustment and channel attention are evaluated through component-wise ablation experiments and the scale-specific average precision. Comparisons with lightweight YOLO models and alternative attention mechanisms are also conducted, and the resulting model is further assessed using an independent test set and a unified batch-size-one latency protocol. The main contributions are summarized as follows:

(1) A task-oriented dual-scale tea bud detector is developed by adjusting the detection-scale configuration of YOLO11n. Removing the P3/8 prediction branch and the corresponding high-resolution feature fusion path reduces the model parameter count and computational complexity while retaining the P4/16 and P5/32 detection scales.
(2) Standard adaptive ECA modules are integrated into the retained P4 and P5 feature paths. Local cross-channel interaction is performed without dimensionality reduction, providing channel response recalibration with a negligible parameter overhead.
(3) The effects of P3 removal and ECA integration are systematically evaluated through ablation experiments and the scale-specific average precision. Independent test-set evaluation, the mAP@0.5:0.95, model complexity analysis, and unified single-image latency measurements are further used to assess the detection performance and computational efficiency of the proposed model.

## 2. Materials and Methods

### 2.1. Dataset Construction

The tea bud images used in this study were provided by the Tea Research Institute of the Chinese Academy of Agricultural Sciences. A total of 526 images were used for model development, including 421 images for training and 105 images for validation, corresponding to an approximate ratio of 4:1. The training and validation sets contained 3337 and 813 annotated tea bud instances, respectively. All images were stored in .jpg format with consistent image dimensions.

**Figure 1:**
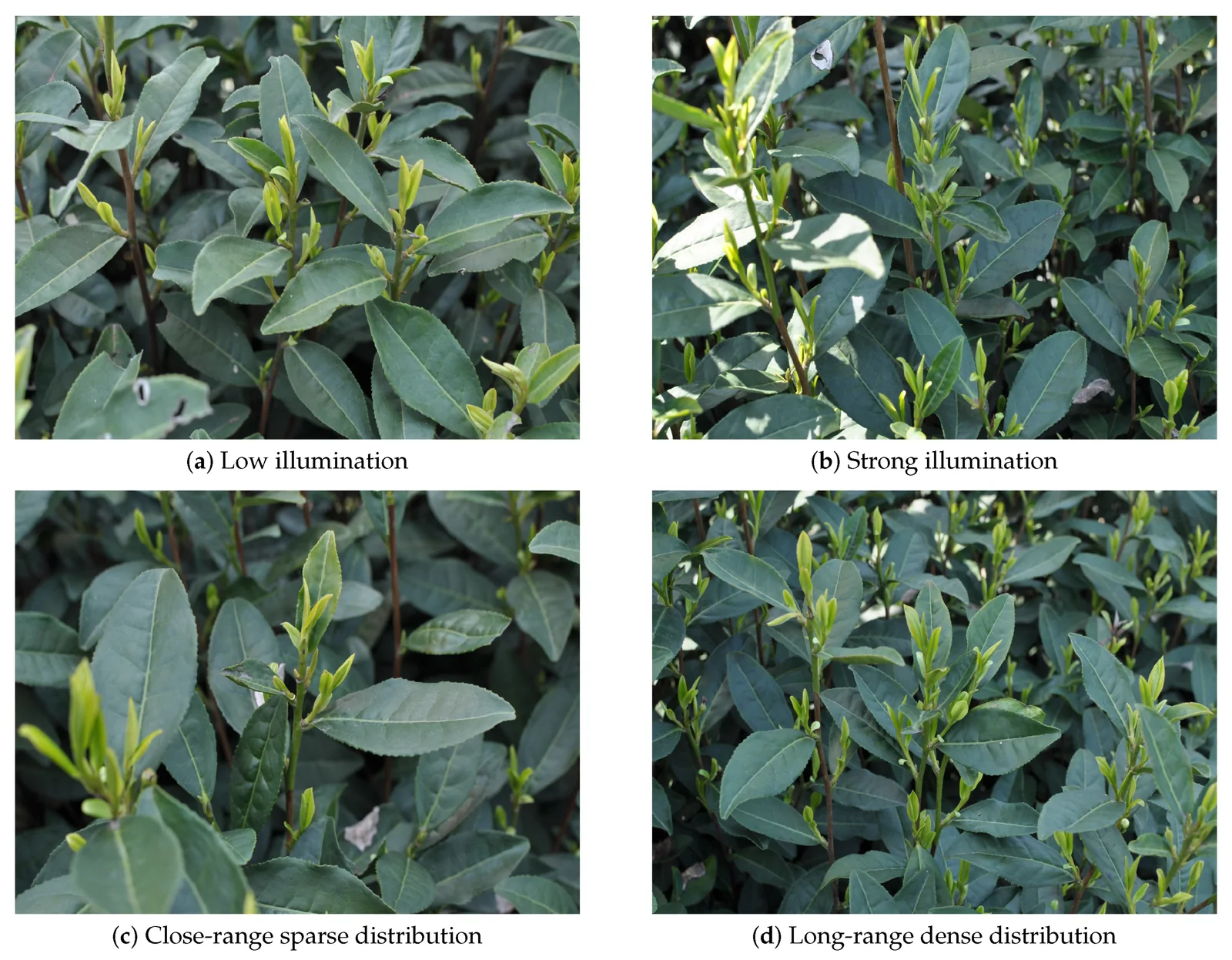
Representative tea bud images under different plantation conditions.

The dataset included images acquired at different viewing distances and camera angles, under varying illumination, occlusion, and target density conditions. Representative samples are shown in Figure 1, including low- and strong-illumination scenes, as well as close-range sparse and long-range dense distributions. These scenes reflect common visual challenges in natural tea plantations, such as uneven illumination, interference from surrounding leaves, target overlap, and variations in apparent target size.

All tea bud targets were manually annotated using LabelImg. Single buds, one-bud-one-leaf shoots, and one-bud-two-leaf shoots were treated as a single category, termed “tea bud”. Bounding boxes were drawn around the tender shoot body while minimizing the inclusion of the surrounding background and mature leaves. When an adjacent tender leaf was closely connected to the shoot and could not be clearly separated, a small portion of the leaf was retained within the bounding box. Targets were excluded when more than approximately 50% of the target was occluded and the bud tip or other defining shoot structures could not be reliably identified. After the initial annotation, the labels were reviewed by another annotator to check for missing targets, inaccurate bounding boxes, and invalid annotations.

In addition to the training and validation sets, an independent test set containing 16 images and 219 annotated tea bud instances was prepared. The independent test images were not used for model training, architecture design, hyperparameter selection, or best checkpoint selection. The same annotation criteria were applied to the training, validation, and independent test sets. Image comparison confirmed that there were no duplicate images among the three subsets.  

### 2.2. Baseline YOLO11n Architecture

**Figure 2:**
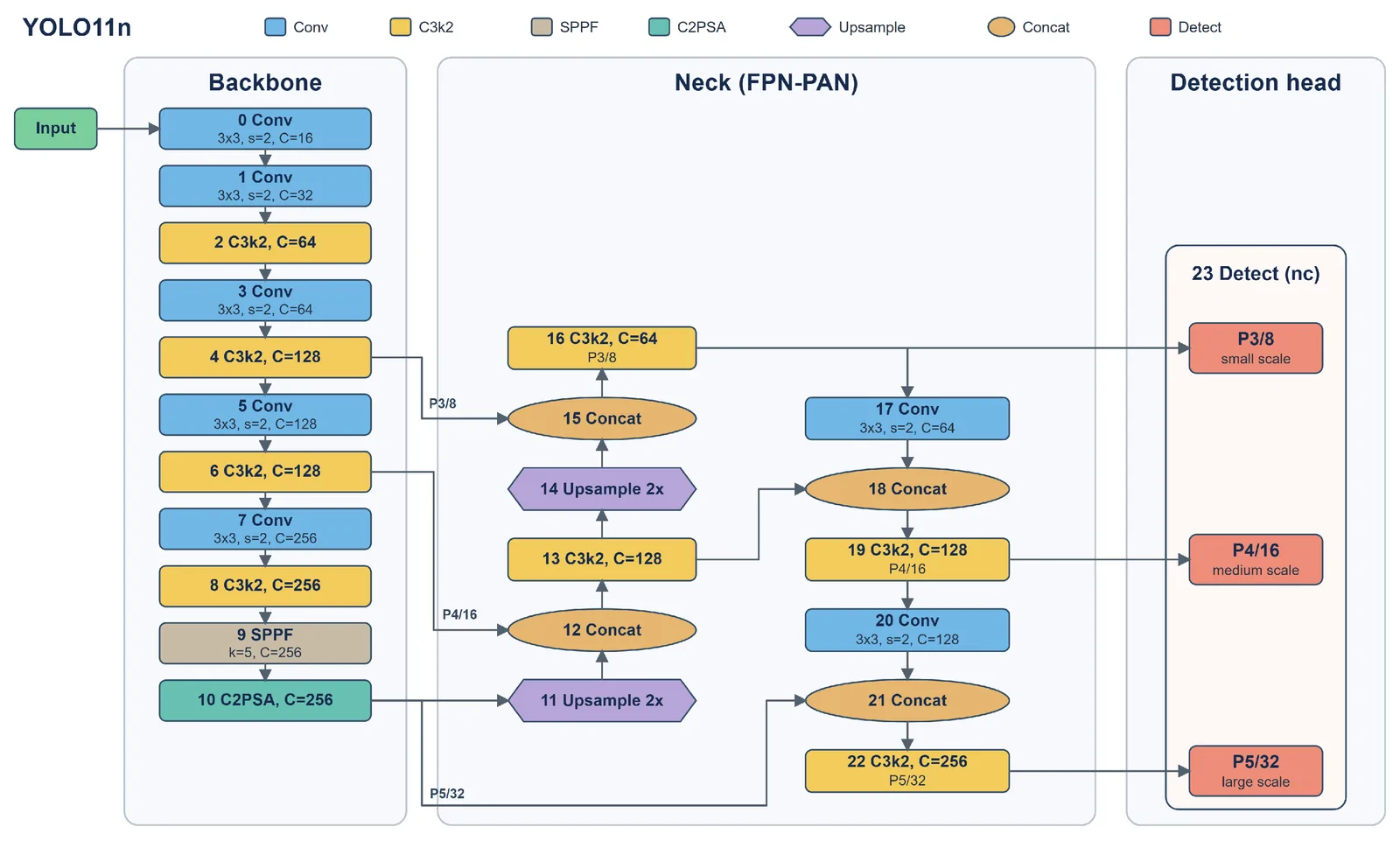
Architecture of the baseline YOLO11n model.

As a representative single-stage object detection framework, the YOLO series integrates object classification and bounding box regression into an end-to-end network [12]. Unlike two-stage detectors, it eliminates the separate region proposal stage and directly produces predictions from multi-scale feature maps, thereby simplifying the detection pipeline and improving the inference efficiency. YOLO11 [13] follows this detection paradigm and consists of a backbone, a neck, and an anchor-free detection head, as illustrated in Figure 2. The backbone employs convolutional layers and C3k2 modules to extract hierarchical features, followed by the Spatial Pyramid Pooling Fast (SPPF) and C2PSA modules for high-level feature processing. The neck aggregates features from different backbone stages through top-down and bottom-up propagation paths, following the general principles of feature pyramid and path aggregation networks [14,15], and the resulting multi-scale features are delivered to the detection head for object classification and bounding box regression.

To accommodate different computational budgets, the YOLO11 family provides several model scales, including YOLO11n, YOLO11s, YOLO11m, YOLO11l, and YOLO11x. Tea bud detection systems intended for field operation may be constrained by the computing capacity, memory, and power consumption of agricultural equipment. Since this study focuses on improving the detection performance without increasing model complexity, YOLO11n was selected as the baseline. As the smallest variant in the YOLO11 family, it provides a low-complexity starting point while retaining the complete feature extraction, feature fusion, and three-scale prediction structure of the original architecture. This configuration allows the effects of detection branch adjustment and ECA integration to be examined without introducing additional network depth or width.

The original YOLO11n model performs prediction at P3/8, P4/16, and P5/32, where the values following the slashes denote the downsampling strides relative to the input image. For an input resolution of 640×640 pixels, the corresponding prediction feature maps have spatial resolutions of 80×80, 40×40, and 20×20, respectively. The P3 branch preserves finer spatial information, whereas the P4 and P5 branches operate on lower-resolution feature maps with progressively larger receptive fields. Under the configuration adopted in this study, the baseline model contains approximately 2.590 million parameters and requires 6.441 GFLOPs. This original three-scale architecture serves as the reference for the subsequent detection scale adjustment and ECA integration.

### 2.3. Proposed YOLO11-ECA

#### 2.3.1. Detection Scale Adjustment

**Figure 3:**
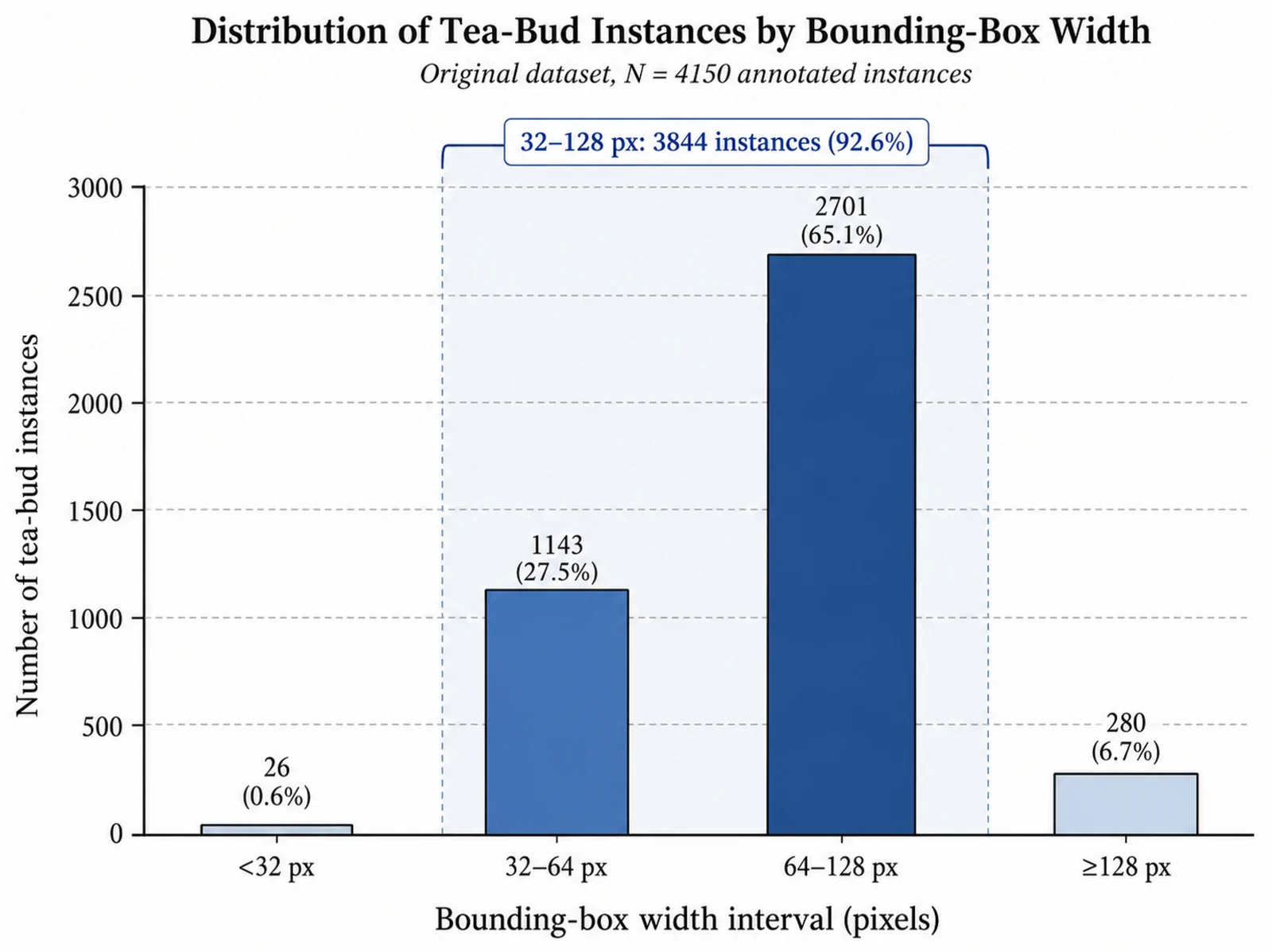
Bounding box width distribution of the annotated tea bud instances in the training and validation sets.

The scale profile of the dataset was characterized using the bounding box widths of the tea bud instances in the training and validation sets. As shown in Figure 3, the two subsets contained 4150 annotated instances. Of these, 1143 instances (27.5%) had widths between 32 and 64 pixels, and 2701 instances (65.1%) had widths between 64 and 128 pixels. These two intervals accounted for 92.6% of all annotations. In comparison, only 26 instances (0.6%) had widths below 32 pixels, while 280 instances (6.7%) had widths of at least 128 pixels. The target distribution was therefore dominated by bounding box widths in the range of 32–128 pixels. These statistics describe the scale composition of the dataset and do not imply a fixed correspondence between individual width intervals and specific feature pyramid levels.

Considering the observed scale composition and the lightweight design requirement, the original three-scale detection structure of YOLO11n was reconfigured as a dual-scale architecture. The P3/8 prediction branch and its associated high-resolution feature fusion path were removed, while the P4/16 and P5/32 branches were retained. Consequently, the detection head performs prediction using feature maps with downsampling strides of 16 and 32. This configuration reduces the high-resolution feature fusion and prediction operations in the neck and detection head, forming the basic lightweight structure of YOLO11-ECA.

#### 2.3.2. Efficient Channel Attention

Efficient channel attention (ECA) is a lightweight channel attention mechanism that captures local cross-channel interactions without dimensionality reduction [16]. In YOLO11-ECA, ECA modules are applied to the retained P4 and P5 feature paths to recalibrate the channel responses before prediction.

**Figure 4:**
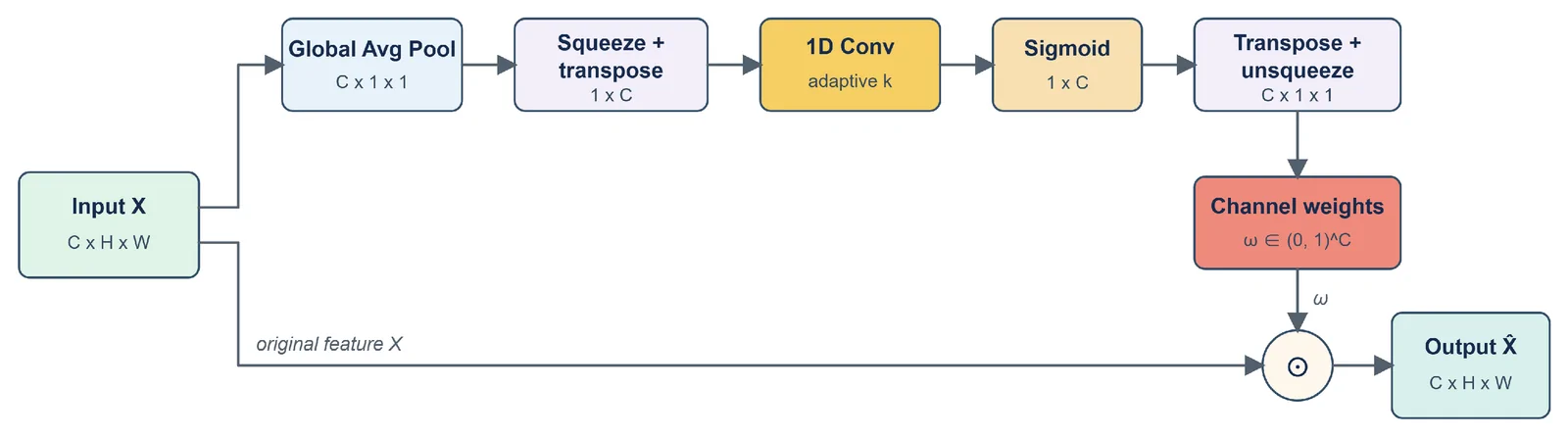
Processing procedure of the ECA module.

Given an input feature map $X\in R^{C\times H\times W}$, global average pooling first aggregates the spatial information of each channel. A one-dimensional convolution is then applied along the channel dimension, and the output is mapped to a channel weight vector through the Sigmoid function:

(1) $$\omega =\sigma \left({\text{Conv1D}}_{k}\left(\mathrm{GAP}(X)\right)\right)$$

where GAP(·) denotes global average pooling, ${\text{Conv1D}}_{k}\left(\cdot \right)$ denotes one-dimensional convolution with kernel size *k*, and $\omega \in R^{C}$ denotes the channel weight vector. The convolution kernel size is adaptively determined by the number of input channels:

(2) $$k={\left|\frac{\log_{2}\left(C\right)+b}{\gamma }\right|}_{\mathrm{odd}}$$

where ${\left|\cdot \right|}_{\mathrm{odd}}$ denotes the mapping of the calculated value to an odd-valued kernel size. Following the original ECA configuration, γ=2 and b=1 were adopted directly without task-specific tuning. In the implementation, the adaptive kernel size was first converted to an integer, and, if the resulting value was even, it was increased by one to obtain an odd kernel size. The calculated weights are broadcast along the spatial dimensions and multiplied channel-wise with the input feature map:

(3) $$\hat{X}=X⊙\omega$$

where ⊙ denotes channel-wise multiplication and $\hat{X}$ is the recalibrated feature map. The P4 and P5 feature paths in the proposed network contain 128 and 256 channels, respectively. According to Equation (2), the kernel size is k=5 for both paths. Each ECA module therefore introduces five trainable parameters. The processing procedure is illustrated in Figure 4.

#### 2.3.3. Overall Network Architecture

**Figure 5:**
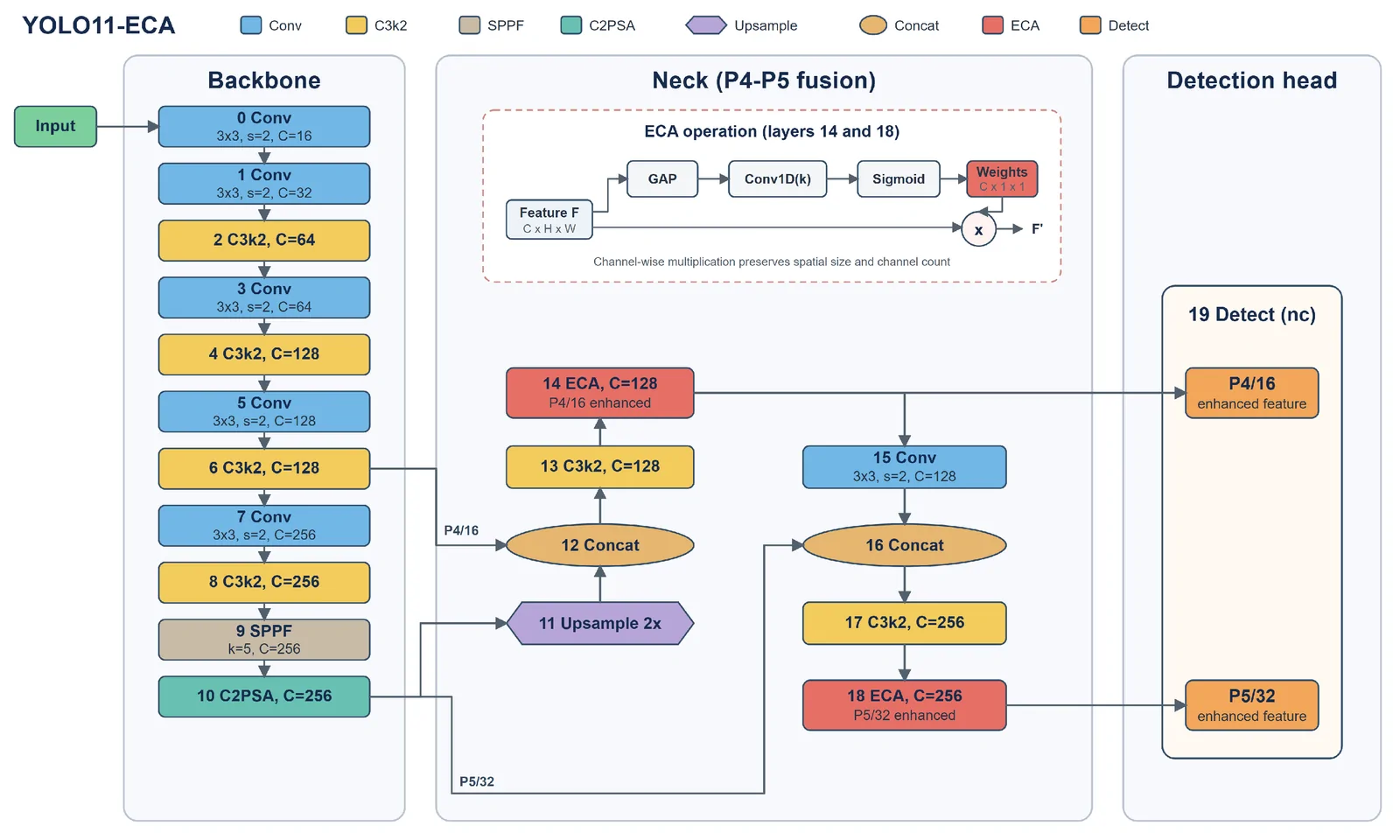
Overall architecture of the YOLO11-ECA model.

The overall architecture of YOLO11-ECA is shown in Figure 5. The backbone retains the original feature extraction structure of YOLO11n, whereas the neck and detection head are reorganized into a two-scale prediction framework by removing the feature fusion pathway and prediction branch associated with P3/8 and retaining P4/16 and P5/32 for detection. Thus, the modification is confined to the subsequent feature aggregation and prediction stages without altering the hierarchical feature extraction performed by the backbone. Specifically, the P5-level backbone feature is upsampled by a factor of two at layer 11 and concatenated with the P4/16 backbone feature at layer 12. After feature fusion through the C3k2 module at layer 13, the resulting feature is recalibrated by the ECA module at layer 14 to form the P4/16 prediction feature. This feature is supplied directly to the Detect head and is simultaneously downsampled by a 3×3 convolution with a stride of 2 at layer 15. The downsampled feature is then concatenated with the P5-level backbone feature at layer 16, followed by further aggregation through the C3k2 module at layer 17 and channel recalibration by the ECA module at layer 18 to generate the P5/32 prediction feature. Consequently, the Detect head receives the outputs of layers 14 and 18 for two-scale prediction. For an input resolution of 640×640 pixels, the P4/16 and P5/32 prediction feature maps have spatial resolutions of 40×40 and 20×20, with 128 and 256 channels, respectively.

In the implemented network, the two ECA modules are inserted at layers 14 and 18, corresponding to the retained P4 and P5 feature pathways. Each module first applies global average pooling to obtain a channel descriptor and then performs local cross-channel interaction using a one-dimensional convolution followed by a sigmoid activation to generate channel-wise weights. The resulting weights are multiplied with the input feature map for channel recalibration. Since ECA does not involve channel reduction or spatial transformation, the spatial resolution and channel dimension of the input features remain unchanged, allowing the modules to be incorporated into the two prediction pathways without additional projection or channel matching operations. The input channel dimensions of the two ECA modules are 128 and 256, respectively, and the adaptive kernel size formulation gives a one-dimensional convolution kernel size of 5 for both modules. Therefore, the two ECA modules introduce only 10 trainable parameters in total. At an input resolution of 640×640 pixels, the resulting YOLO11-ECA contains 2.397 million parameters and requires 4.583 GFLOPs. Compared with the baseline YOLO11n, which contains 2.590 million parameters and requires 6.441 GFLOPs, the parameter count and computational complexity are reduced by approximately 7.4% and 28.8%, respectively. The reduction in model complexity is mainly attributable to the removal of the P3-related high-resolution feature fusion and prediction pathway, while the two ECA modules introduce only limited additional overhead.

### 2.4. Experimental Setup

All experiments were implemented using the Ultralytics 8.3.6 framework with Python 3.10.18, PyTorch 2.1.0, and CUDA 11.8. Model training and evaluation were performed on a computer equipped with an NVIDIA GeForce RTX 3050 laptop GPU with 4 GB of memory. Unless otherwise specified, all models were trained using the same dataset partition, input resolution, optimization schedule, and data augmentation settings. The YOLO11-based models were initialized from the official COCO-pretrained yolo11n.pt checkpoint, while the other comparison models used their corresponding official pretrained weights. Parameters were transferred to layers with compatible structures, whereas newly introduced modules retained their framework initialization.

The input resolution was fixed at 640×640 pixels, with a batch size of 4 and a maximum training duration of 120 epochs. Stochastic gradient descent (SGD) was adopted with an initial learning rate of 0.01, a momentum of 0.937, and a weight decay of 0.0005. Automatic mixed-precision training was enabled to reduce memory consumption and improve computational efficiency. The principal online augmentation operations included Mosaic augmentation, horizontal flipping, HSV color adjustment, random translation, and scale transformation. Mosaic augmentation was disabled during the final 10 epochs so that the model could complete the final optimization stage using images with their original spatial compositions. The main training settings are summarized in Table 1.

Checkpoint selection was based exclusively on validation-set performance. For each model, the checkpoint with the highest validation mAP@0.5:0.95 was retained, and the corresponding precision, recall, mAP@0.5, mAP@0.5:0.95, and F1 score were reported. The independent test set was evaluated only after the network configuration, training parameters, and final checkpoint had been fixed. Therefore, the test images were not involved in model development, hyperparameter adjustment, or checkpoint selection.

Inference efficiency was evaluated on the same RTX 3050 laptop GPU using FP32 precision, a batch size of 1, and an input resolution of 640×640 pixels. Following five warm-up runs, the 16 independent test images were processed over 20 repeated trials. The mean end-to-end latency and frames per second (FPS) were reported, with latency measured from image preprocessing to the final detection output.

### 2.5. Evaluation Metrics

To evaluate the detection accuracy and computational efficiency of the models, the precision, recall, F1 score, mAP@0.5, and mAP@0.5:0.95 were used as the principal performance metrics. The scale-specific average precision was further calculated to examine the detection performance for tea buds of different sizes. In addition, the number of parameters, floating-point operations, end-to-end latency, and frames per second were used to assess model complexity and inference efficiency.

Precision represents the proportion of correct detections among all tea bud predictions. Higher precision indicates that fewer background regions, such as mature leaves, branches, and other visually similar structures, are incorrectly identified as tea buds. It is calculated as

(4) $$\mathrm{Precision}=\frac{TP}{TP+FP}$$

where TP denotes the number of predicted bounding boxes correctly matched to ground-truth tea buds under the specified intersection over union (IoU) criterion, and FP denotes the number of predictions that are not correctly matched to any ground-truth target.

Recall represents the proportion of ground-truth tea buds that are successfully detected. Higher recall indicates fewer missed targets, which is particularly important when tea buds are partially occluded or distributed densely in field images. Recall is defined as

(5) $$\mathrm{Recall}=\frac{TP}{TP+FN}$$

where FN denotes the number of ground-truth tea buds without a valid matching prediction.

The average precision (AP) summarizes the precision–recall relationship at a specified IoU threshold and is calculated from the area under the interpolated precision–recall curve:

(6) $$AP\left(t\right)=\int_{0}^{1}P_{\mathrm{interp}}\left(R,t\right)dR$$

where $P_{\mathrm{interp}}\left(R,t\right)$ denotes the interpolated precision at recall *R* under the IoU threshold *t*. The mAP@0.5 is obtained at an IoU threshold of 0.5 and reflects the overall ability of the detector to identify and localize tea buds. The mAP@0.5:0.95 averages the AP values calculated at ten IoU thresholds from 0.50 to 0.95 with an interval of 0.05, thereby providing a stricter assessment of the bounding box localization accuracy. Since the detection task contains only one category, the reported mAP values are numerically equal to the corresponding AP values of the tea bud class.

To examine the effects of detection scale adjustment on targets of different sizes, the scale-specific average precision was reported as $AP_{S}$, $AP_{M}$, and $AP_{L}$. Following the COCO area definitions [17], targets with bounding box areas $A<32^{2}$, $32^{2}\leq A<96^{2}$, and $A\geq 96^{2}$ pixels squared were categorized as small, medium, and large objects, respectively. These metrics were evaluated over IoU thresholds ranging from 0.50 to 0.95. Because the independent test set contained no large objects, $AP_{L}$ was not reported for the corresponding scale-specific comparison.

The F1 score is the harmonic mean of the precision and recall and provides a combined measure of false detections and missed detections:

(7) $$F_{1}=2\frac{\mathrm{Precision}\times \mathrm{Recall}}{\mathrm{Precision}+\mathrm{Recall}}$$

A higher F1 score indicates a better balance between precision and recall.

Model complexity was evaluated using the number of parameters and giga floating-point operations (GFLOPs) at an input resolution of 640×640 pixels. Inference efficiency was characterized by the mean end-to-end latency per image and the corresponding frames per second (FPS). A lower parameter count, GFLOPs, and latency indicate lower computational requirements, whereas a higher FPS value represents faster image processing capabilities.

## 3. Results

### 3.1. Comparison with Lightweight YOLO Models

**Figure 6:**
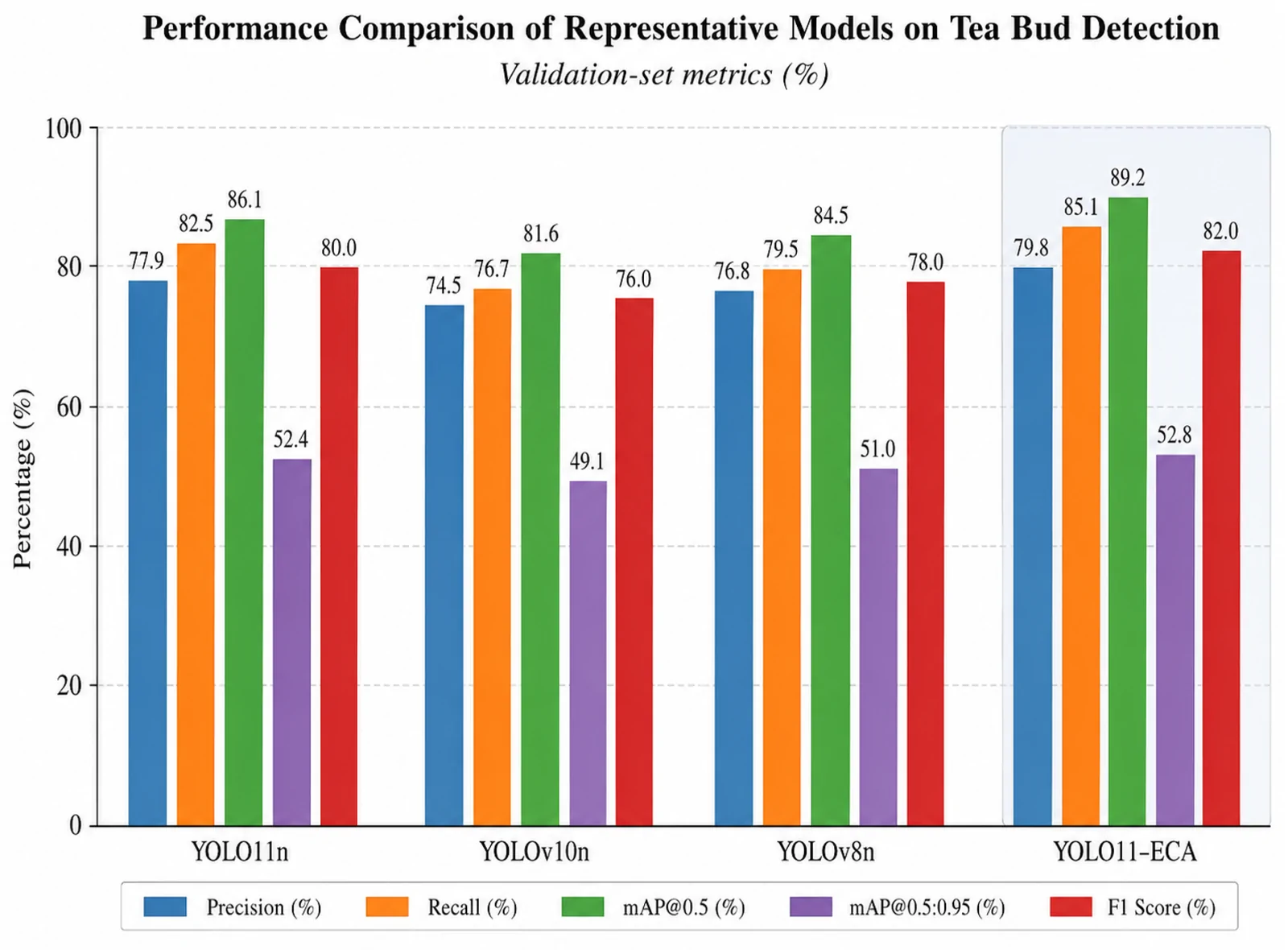
Validation performance comparison of the evaluated lightweight YOLO models.

To evaluate the overall performance of the proposed model, YOLO11-ECA was compared with YOLOv8n [18], YOLOv10n [19], and the baseline YOLO11n under the same dataset partition and training configuration. These models are lightweight variants of their respective YOLO series and have comparable parameter scales. Figure 6 provides a visual comparison of the principal validation metrics, while the complete quantitative results are presented in Table 2.

YOLO11-ECA achieved precision of 79.8%, recall of 85.1%, an mAP@0.5 of 89.2%, an mAP@0.5:0.95 of 52.8%, and an F1 score of 82.0%, obtaining the highest value for each accuracy metric among the evaluated models. Compared with YOLO11n, the precision, recall, mAP@0.5, mAP@0.5:0.95, and F1 score increased by 1.9, 2.6, 3.1, 0.4, and 2.0 percentage points, respectively. The simultaneous improvement in precision and recall resulted in a higher F1 score, indicating a better overall balance between false detections and missed detections. The improvement was more evident for the mAP@0.5 than for the mAP@0.5:0.95, suggesting that the principal gain occurred in overall target detection, whereas the improvement under stricter localization criteria was comparatively limited. In addition, the mAP@0.5 of YOLO11-ECA exceeded those of YOLOv8n and YOLOv10n by 4.7 and 7.6 percentage points, respectively.

YOLO11-ECA contained 2.397372 M parameters and required 4.583373 GFLOPs, representing reductions of approximately 7.4% and 28.8%, respectively, compared with YOLO11n. Its parameter count and computational cost were also lower than those of YOLOv8n and YOLOv10n. These results show that the improvements in recall, F1 score, and mAP@0.5 were achieved while reducing the model complexity, providing a favorable balance between the tea bud detection performance and computational demands.

### 3.2. Comparison of Attention Mechanisms

To compare the suitability of different lightweight attention mechanisms for the current detection task, ECA was replaced with SE [20], CA [21], and CBAM [22] at the same P4 and P5 positions in the dual-scale network. The network architecture, training configuration, and dataset partition were kept consistent across all models. The original YOLO11n was also included as a reference, and the validation results are presented in Table 3.

Among the attention-based variants, YOLO11-SE achieved the highest precision of 81.5%, whereas YOLO11-ECA obtained the highest recall of 85.1%, mAP@0.5 of 89.2%, and mAP@0.5:0.95 of 52.8%. Compared with SE, the ECA configuration showed a decrease of 1.7 percentage points in precision, accompanied by increases of 4.0 percentage points in recall, 0.9 percentage points in the mAP@0.5, and 1.4 percentage points in the mAP@0.5:0.95. CBAM achieved recall of 84.4%, which was close to that of ECA, but its mAP@0.5 and mAP@0.5:0.95 were lower by 1.5 and 1.7 percentage points, respectively. YOLO11-ECA and YOLO11-CBAM achieved the highest F1 scores of 82.0% among the attention-based variants. The results demonstrate that the four attention mechanisms exhibit different trade-offs among false detections, missed detections, and localization accuracy, rather than a consistent advantage across all evaluation metrics.

The attention-based variants had similar computational complexity. YOLO11-ECA contained 2.397372 M parameters and required 4.583373 GFLOPs, representing the lowest values among the four configurations. CA introduced the greatest computational overhead, whereas the complexity differences among ECA, SE, and CBAM were comparatively small. Under comparable model complexity constraints, the higher recall and mAP values obtained by ECA indicate a more favorable balance between detection performance and computational demands for the dual-scale tea bud detection architecture.

### 3.3. Ablation and Scale-Specific Evaluation

Ablation experiments were conducted to evaluate the effects of detection scale adjustment and ECA on model performance. Four configurations were compared under identical training conditions: the original YOLO11n, YOLO11n with the P3 detection branch removed, YOLO11n with ECA alone, and YOLO11-ECA incorporating both modifications. A checkmark in Table 4 and Table 5 indicates that the corresponding modification was applied.

Removing the P3 branch substantially reduced the model complexity while improving the precision, recall, mAP@0.5, and F1 on the validation set. However, the mAP@0.5:0.95 decreased from 52.4% to 51.1%, indicating that the reduction in computational cost was accompanied by a slight loss in localization performance under stricter IoU criteria. The ECA-only configuration achieved the highest precision and mAP@0.5:0.95 among the four ablation settings, but its recall decreased from 82.5% to 78.3%. When P3 removal and ECA were combined, YOLO11-ECA achieved the highest recall of 85.1% and mAP@0.5 of 89.2%. These results indicate that the two modifications did not produce uniform gains across all metrics. Instead, the combined configuration provided a different balance between detection completeness, localization performance, and computational complexity.

The independent test set contained 116 small and 103 medium tea bud instances, with no large instances available for evaluation. Removing the P3 branch reduced APs from 36.0% to 32.4% and APm from 46.9% to 44.5%. The ECA-only configuration achieved an APs value of 34.4% and an APm value of 46.2%. After ECA was incorporated into the dual-scale architecture, APs increased to 37.0%, exceeding both the Delete-P3 configuration and the original YOLO11n. In contrast, APm was 44.2%, remaining below the YOLO11n baseline. These results show that the scale-specific improvement of YOLO11-ECA was mainly reflected in small-object detection rather than being uniform across object sizes.

### 3.4. Independent Test Set Evaluation

To further evaluate model performance on images excluded from model development, the selected checkpoints were directly applied to an independent test set containing 16 images and 219 annotated tea bud instances. The test images were not used for model training, hyperparameter selection, architecture adjustment, or checkpoint selection. All models were evaluated using identical inference settings without retraining, fine-tuning, or threshold adjustment. The quantitative results are presented in Table 6.

YOLO11-ECA achieved precision of 82.2%, recall of 79.9%, an mAP@0.5 of 84.8%, an mAP@0.5:0.95 of 41.7%, and an F1 score of 81.0%. Compared with YOLO11n, these metrics increased by 6.2, 2.7, 4.0, 0.2, and 4.0 percentage points, respectively. The simultaneous improvements in precision and recall resulted in a higher F1 score, indicating a better balance between false detections and missed detections. The increase in the mAP@0.5 was more pronounced than that in the mAP@0.5:0.95, demonstrating that the principal improvement was reflected in overall tea bud detection, whereas the gain under stricter localization criteria was relatively limited.

Among the evaluated models, YOLO11-ECA obtained the highest precision, mAP@0.5, and F1 score. YOLO11-CBAM achieved the highest recall of 80.3%, while YOLO11-CA produced the highest mAP@0.5:0.95 of 42.0%. These results indicate that the attention mechanisms differed in their balances between detection completeness, false-positive control, and localization accuracy. YOLO11-ECA achieved the strongest combined performance in terms of precision, the mAP@0.5, and the F1 score, and its improvements over YOLO11n were maintained on images not involved in model development.

### 3.5. Inference Efficiency

Inference efficiency was evaluated on an NVIDIA GeForce RTX 3050 laptop GPU using FP32 precision, a batch size of 1, and an input resolution of 640×640 pixels. All models were tested with identical confidence, non-maximum suppression, and maximum detection settings. After five warm-up runs, the 16 independent test images were processed repeatedly for 20 rounds. The mean and standard deviation of the preprocessing time, network inference time, postprocessing time, and end-to-end latency are reported in Table 7. The postprocessing time includes prediction processing and non-maximum suppression (NMS).

YOLO11-ECA achieved an average network inference time of 8.47±0.58 ms and end-to-end latency of 11.59±0.75 ms per image, corresponding to 86.28 FPS. Compared with YOLO11n, its network inference time decreased by approximately 18.2%, while the end-to-end latency decreased by 13.4%. The corresponding processing rate increased from 74.73 to 86.28 FPS, representing an improvement of approximately 15.5%. Although YOLO11-ECA required slightly more preprocessing time than YOLO11n, its lower network inference time resulted in the shortest overall latency among the evaluated models.

YOLO11-ECA also outperformed YOLOv8n and YOLOv10n in end-to-end efficiency. Its latency was approximately 8.9% lower than that of YOLOv8n and 20.7% lower than that of YOLOv10n. These results are consistent with the reductions in parameter count and GFLOPs reported previously, demonstrating that the detection scale adjustment reduced the computational demand in practical inference. Under the current GPU-based evaluation protocol, YOLO11-ECA therefore provided the most favorable combination of detection accuracy and processing efficiency among the compared lightweight models.

### 3.6. Qualitative Analysis

Multi-scale tea bud class response maps were generated to further examine the spatial response characteristics of YOLO11n and YOLO11-ECA. The native prediction scales of each model were used: P3/8, P4/16, and P5/32 for YOLO11n and P4/16 and P5/32 for YOLO11-ECA. For both models, the class logits before bounding box decoding were transformed using the sigmoid function, resized to the input-image resolution, and aggregated through a pixel-wise maximum operation. Identical input images, preprocessing procedures, colormaps, overlay parameters, and pairwise shared min–max normalization were applied to ensure a consistent visual comparison.

**Figure 7:**
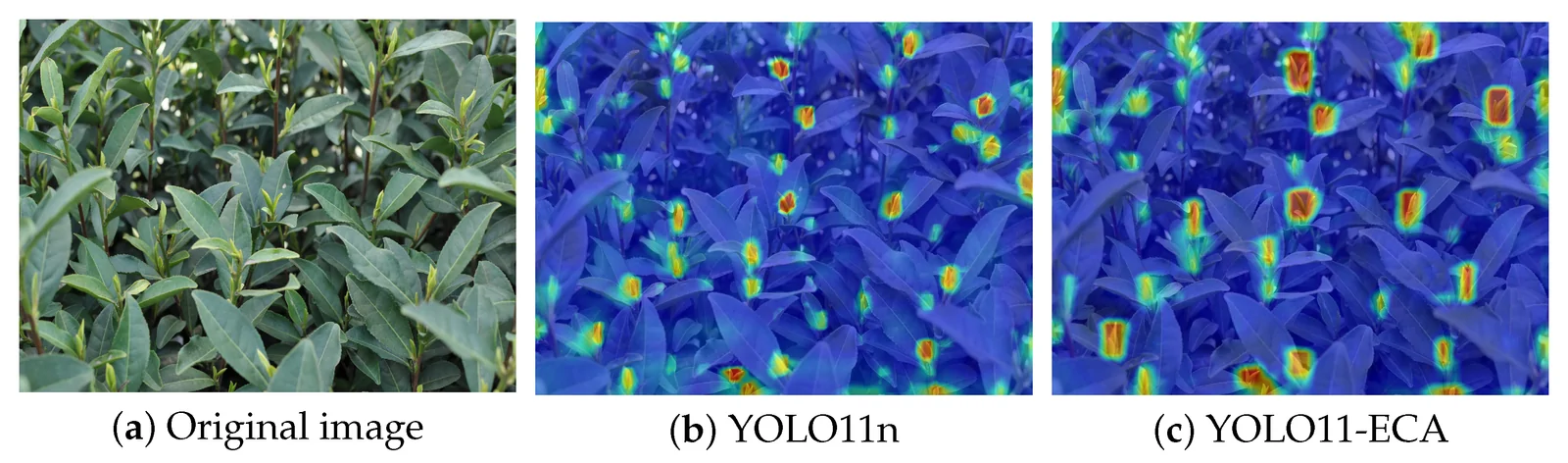
Comparison of tea bud class response distributions under a complex tea canopy scene.

As shown in Figure 7, YOLO11n produced relatively diffuse class responses, with activated regions extending from the tea buds to the surrounding foliage in several areas. In contrast, the responses of YOLO11-ECA were more spatially concentrated around multiple tea bud regions, while weaker activations were observed in portions of the non-target background. This difference indicates that the two models formed distinct spatial response distributions for tea bud features. Nevertheless, activation diffusion remained around heavily occluded and visually ambiguous targets, suggesting that dense leaf overlap and the high visual similarity between tender shoots and surrounding leaves continue to limit feature discrimination.

**Figure 8:**
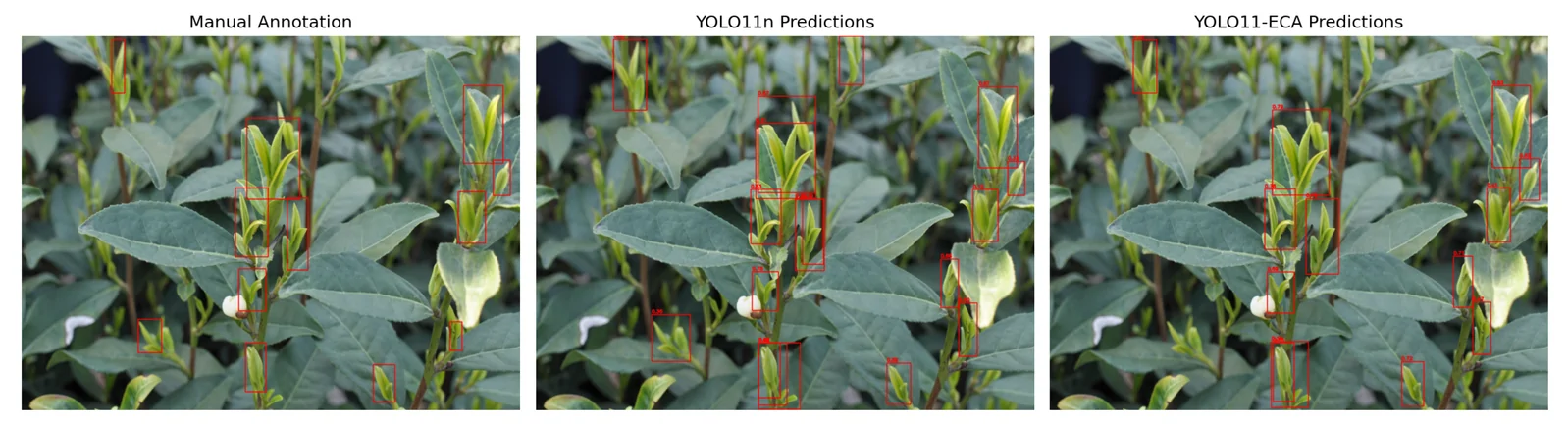
Representative detection comparison among the ground-truth annotation, YOLO11n, and YOLO11-ECA.

Figure 8 further presents a representative detection result in a densely distributed tea canopy scene. The ground-truth annotation contained 12 tea bud instances. YOLO11n generated 18 detection boxes, including several redundant predictions around overlapping leaves and background regions with a tea bud-like appearance, whereas YOLO11-ECA generated 13 detection boxes with a distribution that was more consistent with the annotated targets. Although residual missed and false detections were still present, the reduced number of redundant predictions is consistent with the higher precision obtained by YOLO11-ECA in the quantitative evaluation. The qualitative results therefore complement the numerical comparisons by illustrating the differences between the two models in terms of spatial response concentration and prediction distribution under complex canopy conditions.

## 4. Discussion

Previous tea bud detectors have mainly introduced lightweight convolutions, compact attention modules, feature fusion redesign, or task-specific prediction components to improve the balance between detection performance and the computational cost. In contrast, the lightweight design of YOLO11-ECA was achieved through task-oriented detection scale adjustment rather than the uniform compression of the entire network. Removing the P3 detection branch reduced the parameter count and GFLOPs by approximately 7.4% and 28.9%, respectively, while increasing the precision, recall, mAP@0.5, and F1 on the validation set. However, the mAP@0.5:0.95 decreased from 52.4% to 51.1%, and the scale-specific AP results also declined after P3 removal. This performance pattern indicates that the adjustment of the detection scale changes the allocation of feature representation within the network: eliminating the high-resolution branch reduces the computational demand and improves overall detection at an IoU threshold of 0.5, but also affects scale-sensitive representation and bounding box localization under stricter criteria. For the target size distribution of the current tea bud dataset, the retained P4 and P5 scales therefore provide a task-specific balance between detection performance and computational complexity.

The effect of ECA varied with the detection scale configuration. When ECA was applied to the original three-scale detector, the precision and mAP@0.5:0.95 increased, whereas the recall decreased from 82.5% to 78.3%. This indicates that ECA alone changed the balance between precision and recall, rather than producing a consistent improvement across all detection metrics. When ECA was introduced into the retained P4 and P5 feature paths, YOLO11-ECA achieved the highest recall of 85.1% and mAP@0.5 of 89.2%, while APs increased from 32.4% to 37.0% relative to the Delete-P3 configuration. However, the improvement was not uniform across all metrics or object scales. Comparisons with SE, CA, and CBAM further showed that the effects of different attention mechanisms were metric-dependent. Overall, ECA provided a favorable balance between the detection performance and computational cost in the dual-scale architecture.

The independent test results confirmed that the relative improvement over YOLO11n was maintained on images excluded from model development. YOLO11-ECA increased the precision, recall, mAP@0.5, mAP@0.5:0.95, and F1 by 6.2, 2.7, 4.0, 0.2, and 4.0 percentage points, respectively. The more evident gains in precision, mAP@0.5, and F1 indicate that the improvement was mainly reflected in overall target recognition and false-positive control. In contrast, the limited increase in the mAP@0.5:0.95 shows that accurate bounding box localization under strict IoU thresholds remains an important challenge. The class response maps and representative detection example support this interpretation: YOLO11-ECA produced more concentrated responses around several tea bud regions and reduced redundant predictions in the dense-canopy scene. Nevertheless, overlapping leaves, partial occlusion, and the visual similarity between tender shoots and surrounding foliage continued to cause response diffusion, missed detections, and localization errors.

The reduction in theoretical complexity was also reflected in the practical inference performance. YOLO11-ECA contained 2.397372 M parameters and required 4.583373 GFLOPs, while achieving end-to-end latency of 11.59±0.75 ms and a processing rate of 86.28 FPS under the unified GPU evaluation protocol. The reductions in parameter count and GFLOPs were accompanied by lower measured GPU inference latency under the unified evaluation protocol, indicating that the reduced theoretical complexity was also reflected in practical inference efficiency. This balance between detection performance and processing efficiency demonstrates the practical value of YOLO11-ECA for image-based tea bud monitoring and provides a foundation for subsequent applications such as yield-related analysis and intelligent harvesting. Future research will further extend the model across different cultivars, seasons, acquisition viewpoints, and field environments, while improving feature discrimination and boundary localization under dense and occluded conditions. Deployment-oriented optimization, temporal tracking, repeated-count suppression, and benchmarking on representative edge computing platforms will also be investigated to further improve the robustness and applicability of the method in practical tea production scenarios.

## 5. Conclusions

A lightweight tea bud detection model, YOLO11-ECA, was developed by removing the P3 detection branch and introducing ECA into the retained P4 and P5 feature paths. The dual-scale design reduced the parameter count and GFLOPs by approximately 7.4% and 28.8%, respectively, compared with YOLO11n. On the validation set, the proposed model achieved recall of 85.1%, an mAP@0.5 of 89.2%, and an mAP@0.5:0.95 of 52.8%. The scale-specific evaluation showed that ECA increased $AP_{s}$ from 32.4% for the Delete-P3 configuration to 37.0%, thereby mitigating the reduction in small-object detection performance caused by removing the high-resolution detection branch.

On the independent test set, YOLO11-ECA achieved precision of 82.2%, an mAP@0.5 of 84.8%, and an F1 score of 81.0%, maintaining its overall performance improvement over YOLO11n. The model also reached 86.28 FPS with end-to-end latency of 11.59±0.75 ms under the unified GPU evaluation protocol. These results demonstrate that task-oriented detection scale adjustment combined with lightweight channel attention can improve tea bud detection while reducing the computational demand, providing an efficient visual detection framework for tea bud monitoring and a foundation for subsequent applications such as intelligent harvesting and yield-related analysis.

## Figures and Tables

**Table 1:** Main training settings used in the experiments.

Parameter	Setting
Input resolution	640×640 pixels
Training epochs	120
Batch size	4
Optimizer	SGD
Initial learning rate	0.01
Momentum	0.937
Weight decay	0.0005
Automatic mixed precision	Enabled
Mosaic augmentation	Enabled and disabled during the final 10 epochs
HSV augmentation	h=0.015,s=0.7,v=0.4
Translation	0.1
Scale transformation	0.5
Horizontal flipping	0.5
Checkpoint criterion	Highest validation mAP@0.5:0.95

**Table 2:** Validation-set comparison of lightweight YOLO models. The best value in each column is shown in bold.

Model	Precision (%)	Recall (%)	mAP@0.5 (%)	mAP@0.5:0.95 (%)	F1 (%)	Parameters (M)	GFLOPs
YOLOv10n	74.5	76.7	81.6	49.1	76.0	2.707430	8.392960
YOLOv8n	76.8	79.5	84.5	51.0	78.0	2.690403	6.942259
YOLO11n	77.9	82.5	86.1	52.4	80.0	2.590035	6.440602
YOLO11-ECA	**79.8**	**85.1**	**89.2**	**52.8**	**82.0**	**2.397372**	**4.583373**

**Table 3:** Validation-set comparison of different attention mechanisms. The best value in each column is shown in bold.

Model	Precision (%)	Recall (%)	mAP@0.5 (%)	mAP@0.5:0.95 (%)	F1 (%)	Parameters (M)	GFLOPs
YOLO11n	77.9	82.5	86.1	52.4	80.0	2.590035	6.440602
YOLO11-ECA	79.8	**85.1**	**89.2**	**52.8**	**82.0**	**2.397372**	**4.583373**
YOLO11-SE	**81.5**	81.1	88.3	51.4	81.0	2.398130	4.583680
YOLO11-CA	80.7	81.7	87.4	51.7	81.0	2.407394	4.597453
YOLO11-CBAM	79.8	84.4	87.7	51.1	**82.0**	2.398326	4.584686

**Table 4:** Ablation results on the validation set. The best value in each column is shown in bold.

Model	P3 Removal	ECA	Precision (%)	Recall (%)	mAP@0.5 (%)	mAP@0.5:0.95 (%)	F1 (%)	Parameters (M)	GFLOPs
YOLO11n	–	–	77.9	82.5	86.1	52.4	80.0	2.590035	6.440602
Delete-P3	✓	–	81.7	83.6	87.1	51.1	**83.0**	**2.397362**	**4.582144**
ECA-only	–	✓	**82.1**	78.3	87.4	**53.5**	80.0	2.590045	6.441830
YOLO11-ECA	✓	✓	79.8	**85.1**	**89.2**	52.8	82.0	2.397372	4.583373

“✓” indicates that the corresponding operation was performed, while “–” indicates that it was not performed.

**Table 5:** Scale-specific AP results on the independent test set. The best value in each column is shown in bold.

Model	P3 Removal	ECA	${\mathrm{AP}}_{s}$ (%)	${\mathrm{AP}}_{m}$ (%)	${\mathrm{AP}}_{l}$ (%)
YOLO11n	–	–	36.0	**46.9**	–
Delete-P3	✓	–	32.4	44.5	–
ECA-only	–	✓	34.4	46.2	–
YOLO11-ECA	✓	✓	**37.0**	44.2	–

“✓” indicates that the corresponding operation was performed, while “–” indicates that it was not performed.

**Table 6:** Performance comparison on the independent test set. The best value in each column is shown in bold.

Model	Precision (%)	Recall (%)	mAP@0.5 (%)	mAP@0.5:0.95 (%)	F1 (%)
YOLOv10n	69.3330	68.0365	73.9485	37.1169	69.0
YOLOv8n	75.1380	74.5205	78.9251	40.9803	75.0
YOLO11n	76.0092	77.1689	80.8052	41.5103	77.0
Delete-P3	78.9560	78.5388	82.2202	41.6083	79.0
ECA-only	75.6864	78.1787	80.8820	40.7059	77.0
YOLO11-SE	77.6268	77.6256	82.6785	40.9929	78.0
YOLO11-CA	78.2639	78.5388	82.4734	**42.0470**	78.0
YOLO11-CBAM	79.9964	**80.3015**	83.4427	41.8981	80.0
YOLO11-ECA	**82.2187**	79.9087	**84.7727**	41.6944	**81.0**

**Table 7:** Inference efficiency of the lightweight detection models on the independent test set. Timing values are reported as mean ± standard deviation.

Model	Preprocessing (ms)	Inference (ms)	Postprocessing (ms)	End-to-End (ms)	FPS
YOLOv10n	1.81±0.44	12.25±1.92	0.55±0.50	14.61±2.14	68.47
YOLOv8n	1.88±0.42	9.48±1.82	1.37±0.56	12.72±2.26	78.60
YOLO11n	1.83±0.42	10.36±1.55	1.19±0.44	13.38±1.92	74.73
YOLO11-ECA	2.00±0.38	8.47±0.58	1.12±0.34	11.59±0.75	86.28

Evaluation settings: FP32 precision, batch size 1, input size 640×640, confidence threshold 0.25, NMS IoU threshold 0.70, maximum detections 300, five warm-up runs, and 20 repeated evaluations of 16 test images.

## Data Availability

The datasets supporting the findings of this study are available from the corresponding author on reasonable request.

## Abbreviations

The following abbreviations are used in this manuscript:

AMP	Automatic Mixed Precision
AP	Average Precision
CA	Coordinate Attention
CBAM	Convolutional Block Attention Module
CUDA	Compute Unified Device Architecture
ECA	Efficient Channel Attention
FN	False Negative
FP	False Positive
FP32	32-Bit Floating-Point Format
FPN	Feature Pyramid Network
FPS	Frames Per Second
GFLOPs	Giga Floating-Point Operations
GPU	Graphics Processing Unit
HSV	Hue, Saturation, and Value
IoU	Intersection over Union
mAP	Mean Average Precision
NMS	Non-Maximum Suppression
PAN	Path Aggregation Network
SE	Squeeze-and-Excitation
SGD	Stochastic Gradient Descent
TP	True Positive
YOLO	You Only Look Once
